# The conserved GTPase HflX is a ribosome splitting factor that binds to the E-site of the bacterial ribosome

**DOI:** 10.1093/nar/gkv1524

**Published:** 2016-01-04

**Authors:** Mackenzie L. Coatham, Harland E. Brandon, Jeffrey J. Fischer, Tobias Schümmer, Hans-Joachim Wieden

**Affiliations:** Alberta RNA Research and Training Institute, Department of Chemistry and Biochemistry, University of Lethbridge, Lethbridge, Alberta, T1K 3M4, Canada

## Abstract

Using a combination of biochemical, structural probing and rapid kinetics techniques we reveal for the first time that the universally conserved translational GTPase (trGTPase) HflX binds to the E-site of the 70S ribosome and that its GTPase activity is modulated by peptidyl transferase centre (PTC) and peptide exit tunnel (PET) binding antibiotics, suggesting a previously undescribed mode of action for these antibiotics. Our rapid kinetics studies reveal that HflX functions as a ribosome splitting factor that disassembles the 70S ribosomes into its subunits in a nucleotide dependent manner. Furthermore, our probing and hydrolysis studies show that the ribosome is able to activate trGTPases bound to its E-site. This is, to our knowledge, the first case in which the hydrolytic activity of a translational GTPase is not activated by the GTPase activating centre (GAC) in the ribosomal A-site. Furthermore, we provide evidence that the bound state of the PTC is able to regulate the GTPase activity of E-site bound HflX.

## INTRODUCTION

Translation is an essential ribosome-mediated process in all cell types that occurs in four sequential phases: initiation, elongation, termination and recycling. For efficient polypeptide synthesis, additional ribosome associated proteins are required at each of these phases. Several of the involved proteins function as guanosine 5′-triphosphatases (GTPases), utilizing the hydrolysis of GTP to drive their functional cycle. These factors include the canonical and essential translation factors initiation factor (IF) 2, elongation factors (EFs) Tu and G, and release factor (RF) 3. Each of the previously mentioned GTPases have been characterized to be involved in at least one of the phases of translation, yet some, like EF-G, function during both elongation and recycling. Furthermore, there are additional ribosome-associated GTPases not essential for translation including: the EF-Tu homolog SelB, which is responsible for delivery of selenocysteinyl-tRNA to the elongating ribosome (1), the EF-G homologs LepA, BipA, and ribosome protection proteins (RPPs) such as Tet(O) and TetM (responsible for reverse translocating the ribosome (2–4), stress response (5,6) and release of tetracycline from the bacterial ribosome (7–10), respectively).

Structures of these translational GTPases (trGTPases) share common structural features mimicking, to various degrees, the structure of tRNA (11–13). For example, the structure of EF-G•GDP shares a common shape with the ternary complex of EF-Tu•GTP•aminoacyl-tRNA (14,15). Furthermore, cryo-electron microscopy (cryo-EM) reconstructions of ribosome-bound EF-Tu•GTP•aa-tRNA (16), EF-G (17,18), LepA (3,4), BipA (19) and Tet(O)/TetM (10,20), indicate a common binding site for the translational GTPases in the ribosomal A-site. The recent crystal structure of EF-Tu•GTP•aa-tRNA bound to the 70S ribosome revealed that this GTPase activation likely occurs through the correct positioning of the catalytic histidine residue at the end of switch II (DxxGH) by A2662 of the sarcin-ricin loop (SRL), allowing a nucleophilic attack by a water molecule on the γ-phosphate of bound GTP (21). This mechanism has been proposed to be shared amongst trGTPases (21).

**Figure 1.**
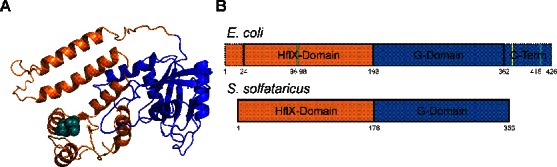
Structural comparison of *Escherichia coli* and *Sulfolobus solfataricus* HflX structures. (**A**) Homology model of *E. coli* HflX using the *S. solfataricus* crystal structure (PDB 2QTH). HflX Is comprised of three domains, an N-terminal HflX-domain unique to HflX (orange) an internal G-domain found in all GTPases (blue), and a C-terminal domain not found in the *S. solfataricus* homolog. Two resolved cysteine residues (teal spheres) are located within the HflX domain in close proximity, while the third cysteine is located in the unresolved C-terminal extension. (**B**) Graphical representation of *E. coli* HflX based on *S. solfataricus* domain layout and length comparison. The C-terminal domain conserved among bacteria but not found in the archaeal species *S. solfataricus*, is shown in dotted blue. Truncation variant of HflX at position 372 indicated by yellow line in *E. coli* layout. The position of cysteines 96, 98 and 415 are indicated by teal lines.

The universally conserved protein HflX (22–24), whose GTPase activity is also enhanced significantly by 50S and 70S ribosomal particles (22), provides an exception to the above common features of trGTPases. The X-ray crystal structure of HflX from *Sulfolobus solfataricus* reveals that the N-terminus of the factor is unique, with no identifiable structural homolog (25). The *Escherichia coli* homolog of HflX is a three-domain protein consisting of the unique N-terminal HflX-domain, a central G-domain and a C-terminal domain not found in the archaeal *S. solfataricus* homolog (Figure 1A). Additionally, *E. coli* HflX has a 22 amino acid N-terminal extension (Figure 1B). These extensions at the termini of HflX are not unique to the *E. coli* protein, but are found in most bacteria and eukaryotes at varying lengths. Several studies have studied truncations of these domains and found reduced binding to the ribosome and differences in nucleotide preference (26). Furthermore, knockout strains of HflX are viable, yet are more susceptible to high intercellular levels of manganese (27). In *E. coli* the gene encoding HflX is found downstream of the gene for Hfq, the universal stress response protein in bacteria, and both are under the control of a heat sensitive promoter (28,29).

In an effort to elucidate the binding site of HflX on the ribosome, we conjugated the factor with the cysteine-specific UV-inducible crosslinking reagent 4-Azidophenacyl bromide (AzP), similar to experiments performed with EF-G (30). Mass spectrometric analysis of crosslinks formed between HflX and ribosomal particles upon exposure to UV light revealed the presence of peptides mapped to ribosomal proteins L2, L5 and S18 near the ribosomal E-site. These data suggest that GTPase activation of HflX by the 50S ribosomal subunit could occur via a different mechanism compared to the canonical translational GTPases. To investigate this further, we examined the effect of numerous 50S- and 30S-specific antibiotics on the ribosome-stimulated GTPase activity of HflX: chloramphenicol, a peptidyl transferase centre (PTC) antibiotic, has previously been shown to inhibit this activity (22). Interestingly, additional antibiotics (azithromycin, erythromycin, lincomycin and clindamycin) that bind to the PTC or the peptide exit tunnel (PET) also inhibit the ribosome-stimulated GTPase activity of HflX without affecting ribosome binding. Inhibitors of EF-G (fusidic acid (31)) and EF-Tu (tetracycline (32) and streptomycin (33)) had no effect on the ribosome-stimulated GTPase activation of HflX. Finally, while attempting to determine a 3D structure of HflX bound to the ribosome we discovered that HflX was able to split the 70S ribosome. Using light scattering in conjunction with stopped-flow rapid kinetics, we show that HflX is able to split the ribosome in a nucleotide-dependent manner, similar to that of EF-G and RRF during ribosome recycling (34). The inclusion of HflX in a heat stress response operon downstream of the universal bacterial stress response protein Hfq (28,29), suggests that this function of HflX may also be critical to the cell's response to stress.

## MATERIALS AND METHODS

### Plasmids

The pET28a plasmid encoding HflX from *E. coli* genomic DNA was previously constructed (22) for purification of wild-type HflX via an N-terminal His_6_-tag. Plasmids containing the HflX C415L and HflX ΔL372 mutants were created using the QuikChange II site-directed mutagenesis protocol (Agilent Technologies) and primers that replaced the codon of Cys415 with a leucine and Leu372 with an amber stop codon. The resulting plasmids were sequence verified and called pET*hflX*-C415L and pET*hflX*-ΔL372.

### Purification of HflX and ribosomes

Vacant ribosomes were purified from *E. coli* MRE600 cells as described in Rodnina *et al*. (35). Wild-type HflX, HflX C415L and HflX ΔL372 were all purified as described in Shields *et al*. (22).

### Covalent crosslinking

HflX was covalently linked to the cysteine-specific crosslinker 4-Azidophenacyl bromide. A total of 100 μM HflX was incubated with 300 μM AzP at 4°C for 24 h. The sample was then dialyzed against TAKM_7_ high salt buffer (50 mM Tris-Cl pH 7.5 at 4°C, 70 mM NH_4_Cl, 600 mM KCl, 7 mM MgCl_2_) overnight to remove unreacted AzP. Small aliquots were flash frozen and stored at −80°C prior to use. Complexes were formed in 20 μl volumes (100 pmol AzP-HflX, 20 pmol ribosomes, with or without 10 000 pmol guanine nucleotide) for 15 min at 37°C for 15 min in TAKM_7_ buffer. Samples were then briefly placed on ice for 5 min and diluted to 500 μl with TAKM_7_ before being subjected to microfiltration (see *Microfiltration* below) until 20 μl remained. A total of 10 μl samples were placed in 96 well microtiter plates and exposed to 365 nm UV light (Spectroline model ENF-280C UV light) placed 1 cm above the sample for 15 min at 4°C. Crosslinking reactions were analyzed on 12% SDS-PAGE gels. For the additional bands observed in the presence of UV light after resolving the samples by SDS-PAGE, the gel corresponding to the different protein species were excised, individually de-stained in 100 mM NH_4_HCO_3_/acetonitrile (50:50) and subsequently digested at 37°C for 16 h. Tryptic peptides were first extracted from the gel and partially dried under vacuum to remove acetonitrile and then suspended in 5% acetonitrile and 1% formic acid. Peptide samples were analyzed using an Agilent 1200 nano-HPLC coupled to a LCQ Deca ion trap mass spectrometer (Thermo Scientific). Nanoflow chromatography and electrospray ionization were accomplished by using a PicoFrit fused silica capillary column (ProteoPepII, C18) with 100 μm inner diameter (300 Å, 5 μm, New Objective) and run at 500 nl/min using a 0–45% 45 min linear acetonitrile (with 0.2% formic acid) gradient. Data dependent analysis was performed on the LCQ Deca at a *m*/*z* range of 400–2000. The three most intense multiply charged ions were sequentially fragmented by using collision induced dissociation. After two fragmentations all precursors selected for dissociation were dynamically excluded for 60 s. The resultant data were analyzed using an in-house MASCOT server. Protein mass spectrometry was performed at the Institute for Biomolecular Design at the University of Alberta.

### Primer extension

rRNA was isolated from HflX-AzP complexes with 70S ribosomes that had been exposed to UV light, as described above. The rRNA was isolated by phenol-chloroform extraction of complexes after 15 min of UV light exposure. Initially 1 pM [^32^P] 5′ end-labeled oligonucleotide primer and 10 μg isolated RNA were incubated at 65°C for 5 min and cooled to 47°C for 10 min to allow for denaturation and primer annealing. To the reaction 0.4 μM dNTPs, 10 mM DTT and 5 U AMV Reverse Transcriptase (NEB) were added and extension was carried out at 47°C for 45 min followed by 15 min at 70°C to denature the reverse transcriptase. Samples were ethanol precipitated and analyzed on an 8% acrylamide 8 M urea slab gel using a BioRad Sequi-Gen GT Sequencing cell (BioRad). Gels were dried, exposed to general purpose storage phosphor screen (GE Healthcare) and scanned on a Typhoon (GE Healthcare).

### Microfiltration

Complexes of HflX with the ribosome were formed by incubating 5 μM of wild-type or variant HflX with 1 μM of 70S in the presence of 1 mM GDP or GDPNP for 15 min at 37°C. Incubation on ice for 5 min preceded dilution of samples to 500 μl of TAKM_7_ (50 mM Tris-Cl pH 7.5 at 4°C, 70 mM NH_4_Cl, 30 mM KCl, 7 mM MgCl_2_) buffer and centrifugation for 5 min at 10 000x*g* in Vivaspin-500 columns with a molecular weight cut off (MWCO) of 100 kDa (GE Healthcare). Once a final volume of 20 μl was reached, the samples were diluted a second time to 500 μl and refiltered. The binding of HflX to ribosomes or ribosomal subunits was analyzed using 12% SDS-PAGE gels. Experiments containing antibiotics had respective antibiotic added to a final concentration of 500 μM in both the reaction and subsequent dilution buffer.

### GTPase assays

The release of [^32^P]-labeled inorganic phosphate (P_i_) from [γ-^32^P] GTP (Perkin-Elmer) was monitored to determine the rate of GTP hydrolysis by HflX. To ensure that nucleotides were in their triphosphate form and multiple turnover experiments would not be inhibited by nucleotide diphosphates, [γ-^32^P] GTP (∼100 dpm/pmol) was incubated with 0.25 μg/μl pyruvate kinase (PK), and 3 mM phosphenolpyruvate (PEP) for 15 min at 37°C. Similarly, 15 μM HflX was also incubated with PK and PEP. Reactions were composed of 1 μM HflX, 125 μM radiolabeled nucleotide solution, 1 μM ribosomes or ribosomal subunits and carried out in TAKM_7_ buffer. At different time points, 5 μl of reactions were removed and quenched in 50 μl of 1 M HClO_4_ with 3 mM K_2_HPO_4_. Inorganic phosphate was extracted as a phosphate-molybdate complex following the addition of 300 μl of 20 mM Na_2_MoO_4_ and 400 μl of isopropyl acetate, mixing for 30 s and centrifugation at 17 000x*g* for 5 min. Half of the organic phase (200 μl) containing the radiolabeled inorganic phosphate was removed, added to 2 ml of scintillation cocktail (MP EcoLite) and counted in a Perkin-Elmer Tri-Carb 2800TR liquid scintillation counter. The obtained activity was converted to pmol of liberated inorganic phosphate using the specific activity of the [γ-^32^P] GTP solution and divided by the respective volumes to yield the concentrations. The hydrolysis of GTP independent of enzyme in solution or hydrolysis due to ribosomes alone was subtracted, in order for the concentration of [^32^P_i_] to be determined and plotted as a function of time. The rates of guanine nucleotide hydrolysis by HflX in the presence of ribosomes was obtained by fitting the multiple turnover experiments with a linear equation, where the slope is equal to the apparent rate of nucleotide hydrolysis. Experiments containing antibiotics had respective antibiotic added to a final concentration of 500 μM.

### Light scattering

To monitor the dissociation of 70S ribosomes into 50S and 30S ribosomal subunits, a KinTek SF-2004 Stopped-flow apparatus was utilized. Samples were kept at 20°C, excited at 436 nm and scattering was detected at 90° after passing through 400 nm long-pass cut-off filters. Reactions were performed in TAKM_5_ buffer (50 mM Tris-Cl pH 7.5 at 4°C, 70 mM NH_4_Cl, 30 mM KCl, 5 mM MgCl_2_), by rapidly mixing 2 μM of wild-type or variant HflX in the presence of 250 μM GTP and 0.30 μM of 70S ribosomes. The resulting signals were normalized with respect to the initial light scattering of the solution, setting the initial value as 100% of intact 70S ribosomes. The resulting light scattering traces were first fit with a one- or two-exponential function (Equations (1) and (2)), where k_app_ is the characteristic apparent rate constant, A is the signal amplitude, Ls is the light scattering at time t and Ls_∞_ is the final light scattering signal. Light scattering data were normalized with respect to the initial fit, averaged (5–10 traces typically), and refit with the appropriate equation. Kinetic constants are expressed as the final fit, ±95% confidence interval.

(1) \begin{equation*} {\rm Ls} = {\rm Ls}_\infty + {\rm Aexp}( - {\rm k}_{{\rm app}} {\rm t}) \end{equation*}

(2) \begin{equation*} {\rm Ls} = {\rm Ls}_\infty + {\rm A}_1 {\rm exp}( - {\rm k}_{{\rm app}1} {\rm t}) + {\rm A}_2 {\rm exp}( - {\rm k}_{{\rm app}2} {\rm t}) \end{equation*}

## RESULTS

### HflX binds near the E-site of the 70S ribosome

**Figure 2.**
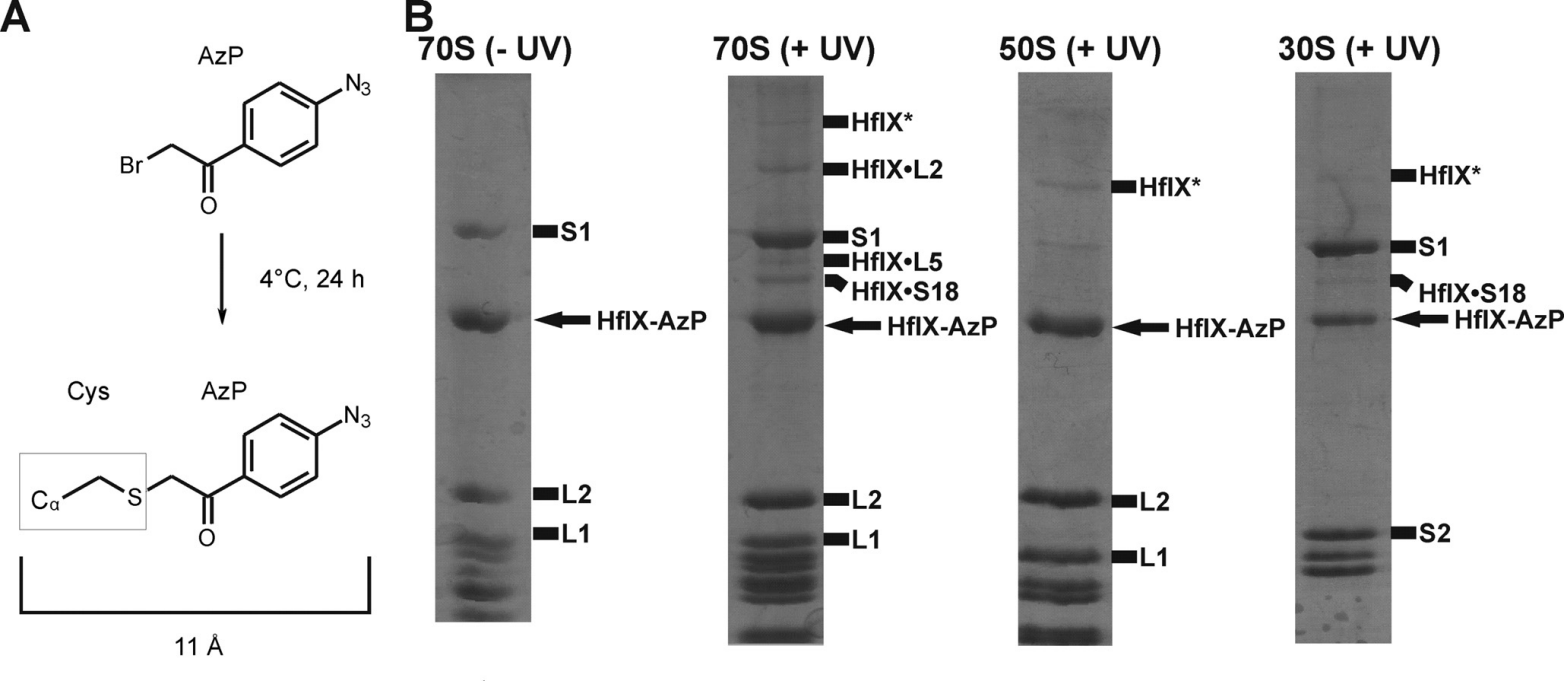
Covalent crosslinking of HflX to the bacterial ribosome. (**A**) HflX was labeled with the cysteine specific crosslinking agent 4-azidophenacyl bromide. (**B**) Complexes of AzP-HflX and 70S ribosomes, or 50S/30S ribosomal subunits (Rb) were formed [5 μM AzP-HflX and 1 μM ribosomal particle] and separated from free AzP-HflX by microfiltration. Resuspended AzP-HflX•Rb complexes were exposed to 365 nm UV light for 15 min before separation of ribosomal proteins by SDS-PAGE. Additional bands of higher molecular weight found in UV-treated samples were excised and sent for mass spectrometry analysis. Bands are annotated with HflX and the respective protein yielding peptides identified by mass spectrometry. Bands marked HflX* only contained peptides identified as HflX derived.

Previous studies have shown that HflX can bind to the 70S ribosome and 50S and 30S ribosomal subunits (36). We employed covalent crosslinking of HflX to the ribosome to determine where on the ribosome HflX binds. To do so, HflX was labeled with the cysteine-specific UV-inducible crosslinker 4-Azidophenacyl bromide (Figure 2A). The AzP crosslinker provides an 11 Å probing radius from labeled cysteine residues. This allows for the identification of proteins and rRNA at or near the binding site of AzP labeled proteins. Three cysteine residues are present in HflX (Figure 1), two at positions 96 and 98 in the N-terminal HflX domain and that are likely to form a disulfide bridge, as well as one at position 415 in the unresolved C-terminal domain.

AzP labeled HflX (AzP-HflX) was incubated with 70S/50S/30S ribosomal particles before microfiltration to remove any unbound HflX before UV exposure to induce crosslinks. Upon exposing the AzP-HflX•ribosome complexes to 365 nm UV light for 15 min, samples were analyzed on SDS-PAGE gels to resolve crosslinks formed (Figure 2B). Additional bands of higher molecular weight than HflX were observed for HflX bound to the 70S ribosome and 50S and 30S ribosomal subunits. These bands were excised and sent for analysis by mass spectrometry. Bands at ∼100, 60 and 56 kDa contained peptides corresponding to ribosomal proteins L2, L5 and S18 (Figure 2B, Supplementary Table S1 and Supplementary Figure S1). Additionally, several bands of lower mobility compared to HflX were observed after UV exposure. These bands only contained peptides derived from HflX (HflX* in Figure 2B), consistent with either inter-HflX or HflX-rRNA crosslinks.

**Figure 3.**
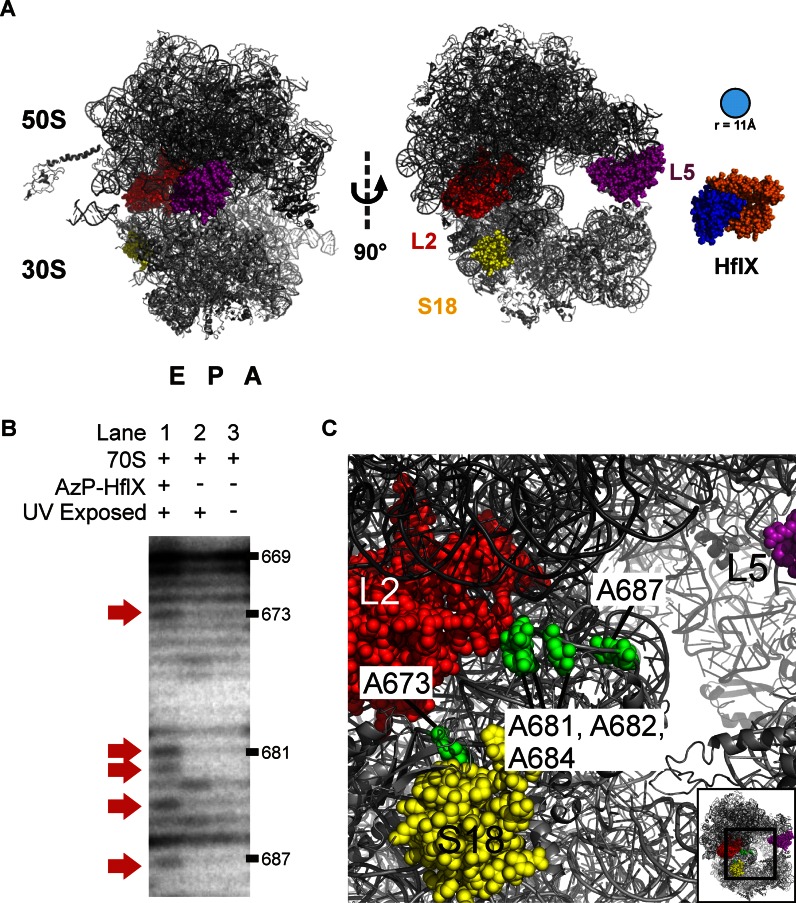
Location of AzP-HflX crosslinks on the bacterial ribosome. (**A**) Protein crosslinks between AzP-HflX and ribosomal proteins L2, L5 and S18 (red, purple and yellow) shown on the bacterial 70S ribosome (PDB 4V4Q). All three ribosomal proteins are on the ribosomal exit-site side of the ribosome and surround the E-site side opening. HflX is shown in blue and orange (PDB 2QTH). (**B**) To confirm the E-site side as the interaction site for HflX, rRNA from AzP-HflX crosslinked ribosome complexes was used in primer extension assays. Using a primer specific for creating cDNA products of helix 23 of the 16S rRNA that lies between ribosomal protein L2 and S18, we were able to find a distinct stopping pattern along the solvent accessible side of helix 23. (**C**) Nucleotides where reverse transcription was halted are shown by green spheres and mapped along the solvent accessible side of helix 23.

These results suggest that HflX binds in or near the ribosomal E-site (Figure 3A). To further confirm this hypothesis, we performed primer extension analysis of rRNA isolated from UV-exposed AzP HflX•70S complexes. Prominent crosslinks to helix 23 of the 16S rRNA were identified by an increased band intensity at nucleotides A673, A681 and A687 (Figure 3B). These nucleotides all lie along the solvent exposed side of the helix. This is consistent with HflX localizing near ribosomal proteins L2 and S18, as helix 23 lies between both proteins in the 70S ribosome (Figure 3C). In addition, the crosslinking pattern observed showed nucleotide dependence, with the *apo* form of HflX yielding the highest crosslinking intensity, followed by GDP and GDPNP (Supplementary Figure S2).

Furthermore, an HflX mutant with the C-terminal cysteine residue replaced with a leucine (HflX C415L) was labeled with AzP. Crosslinking experiments were repeated and analyzed by SDS-PAGE (Supplementary Figure S3). Samples containing AzP-HflX C415L did not contain any visible bands of higher molecular masses compared to wild-type AzP-HflX. This indicates that crosslinks to ribosomal proteins L2, L5, S18 and 16S rRNA helix 23 are within 11 Å of Cys415 in HflX. These results suggest that the C-terminus of HflX is highly flexible and is able to move the minimal distance of 60 Å between L2 and L5.

### Antibiotics targeting the PTC/PET inhibit HflX ribosome-stimulated GTPase activity

**Figure 4.**
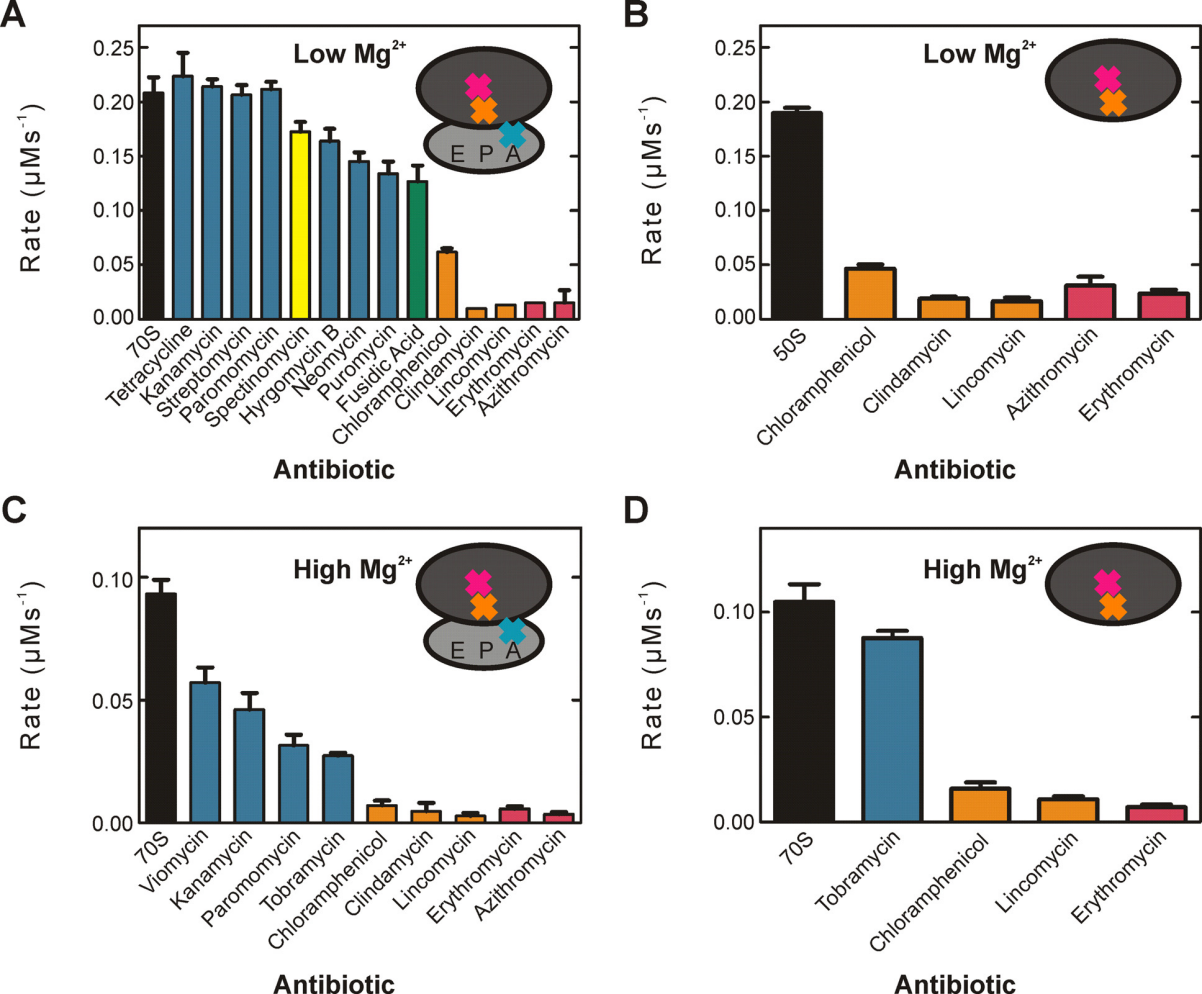
Antibiotic Inhibition of HflX ribosome-stimulated GTP hydrolysis activity. HflX (1 μM) was incubated with (**A**) 70S ribosomes or (**B**) 50S ribosomal subunits (1 μM) in the presence of [γ-^32^P]GTP (125 μM) and antibiotic (500 μM) in low Mg^2+^ buffer (TAKM_5_). Identical reactions for HflX with (**C**) 70S ribosomes and (**D**) 50S ribosomal subunits were carried out in high Mg^2+^ buffer (TAKM_30_). Reactions were quenched at successive time points and the amount of released [^32^P] inorganic phosphate was quantified to determine the rate of hydrolysis. Antibiotics that target several regions of the ribosome were tested including those that bind to the decoding centre (teal), helix 34 near the decoding centre (yellow), EF-G binding site (green), peptidyl transferase centre (orange) and peptide exit tunnel (pink).

To explore which functional centers of the ribosome influence GTP hydrolysis by HflX, we performed GTP hydrolysis experiments in the presence of several ribosome targeting antibiotics. Previous work by our lab revealed that chloramphenicol (Chl), a PTC binding antibiotic, prevents stimulation of the GTPase activity of HflX by the ribosome (22). This led us to test other translation inhibiting antibiotics of several classes and binding sites on the ribosome including PTC antibiotics clindamycin (Cln), and lincomycin (Lin); PET antibiotics azithromycin (Azi) and erythromycin (Ery); decoding center binding antibiotics viomycin (Vio), tetracycline (Tet), paromomycin (Par), hygromycin B (HgB), tobramycin (Tob) and the EF-G specific antibiotic fusidic acid (Fus). Of these antibiotics, only the macrolides (Azi/Ery), lincosamides (Cln/Lin) and chloramphenicol had a dramatic effect on the rate of GTP hydrolysis by HflX (Figure 4). This effect was observed for both 70S and 50S dependent stimulation but not on intrinsic hydrolysis by HflX (Supplementary Figure S4).

**Figure 5.**
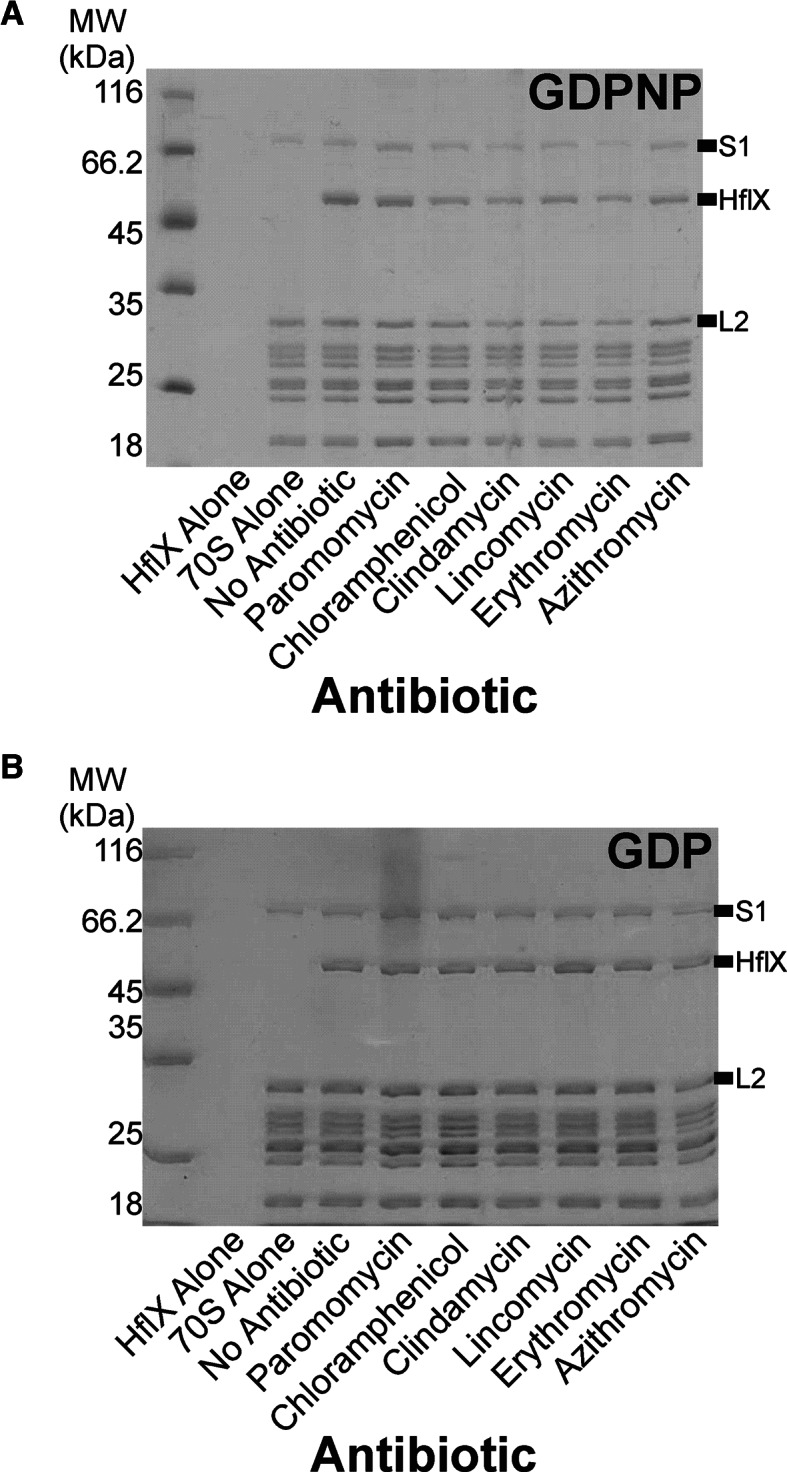
Antibiotic effect on HflX binding to bacterial ribosome. HflX (5 μM) was incubated with 50S ribosomal subunits (1 μM) in the presence of (**A**) GDPNP or (**B**) GDP (500 μM) and the antibiotic indicated (500 μM). Complexes were separated from free HflX via filtration and subsequently washed before collecting the remaining filtrate for analysis by SDS-PAGE.

To determine if the decreased rate of hydrolysis is due to HflX being prevented from binding to its stimulatory partner the ribosome, microfiltration assays were performed in the presence of each antibiotic (Figure 5). Both the GDPNP- (a non-hydrolysable analog of GTP) and GDP-bound states of HflX were examined for binding to the 50S ribosomal subunit by microfiltration and subsequent SDS-PAGE analysis. HflX incubated in the absence of ribosomes was not retained above the filter following filtration, while HflX preincubated with 50S subunits remained above the filter as apparent by a 50.5 kDa band corresponding to the molecular weight of HflX. HflX•50S complexes were incubated with nucleotide (GDP or GDPNP) and antibiotic. No inhibition of HflX binding to the ribosome was observed with any of the antibiotics tested.

Furthermore, to verify that ribosomes were not blocking the filter, and causing the retention of HflX above the filter, we performed the same experiment with the non-ribosome associated protein, EF-Ts (Supplementary Figure S5). EF-Ts, the nucleotide exchange factor for EF-Tu, was not retained above the filter in the presence of ribosomes (Supplementary Figure S5B). Additionally, ultracentrifugation pelleting experiments through 10% sucrose cushions were performed to verify the specificity of HflX binding to the 70S ribosome (Supplementary Figure S6).

### HflX splits the 70S ribosome into 50S/30S ribosomal subunits in a nucleotide-dependant manner

**Figure 6.**
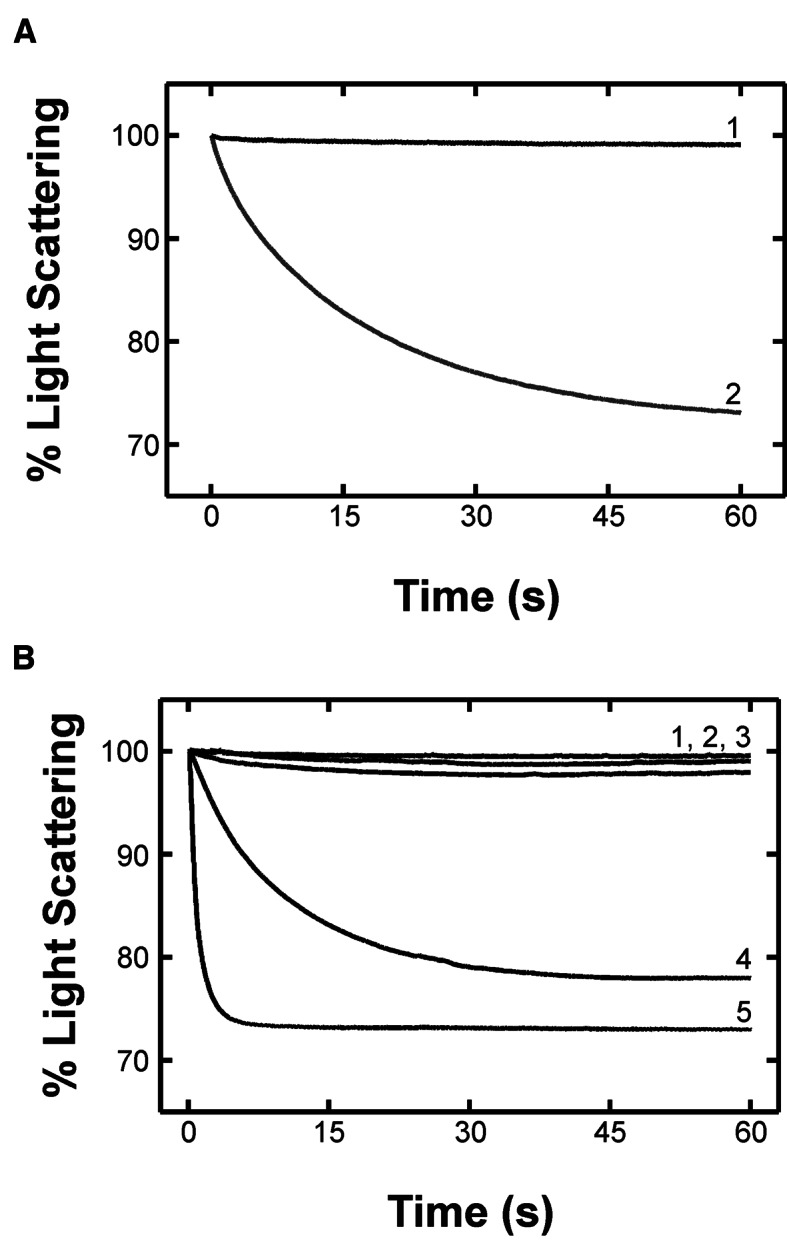
HflX splits the 70S ribosome in a nucleotide dependant manner. Light scattering experiments monitoring the size of particles in solution carried out using a stopped-flow apparatus. Samples are exposed to 426 nm light and scattering is monitored at 90° to the incident light. Larger particles scatter more light than smaller particles. (**A**) 70S ribosomes in TAKM_5_ buffer are mixed with either TAKM_5_ buffer (1) or TAK buffer (2). Decreasing Mg^2+^ concentrations results in 70S dissociation into 50S and 30S subunits. (**B**) 70S ribosomes mixed with TAKM_5_ (1), and HflX in the nucleotide free *apo* state (2), and GDP- (3), GDPNP- (4) or GTP-bound states (5).

Initial experiments on 70S ribosome (0.15 μM final concentration) dissociation (Figure 6A) were performed in a buffer with a final concentration of 2.5 mM Mg^2+^. Under these conditions, 70S ribosomes dissociate completely into 50S and 30S subunits (37). Rapid mixing of 70S ribosomes with buffer lacking Mg^2+^ (2.5 mM final Mg^2+^ concentration after mixing) resulted in a 29% decrease in light scattering intensity with an apparent rate of 0.055 ± 0.001 s^−1^. As a control, 70S ribosomes were mixed with TAKM_5_ buffer (5 mM Mg^2+^ final concentration) resulting in only a small (∼1%) decrease in light scattering intensity. In the presence of HflX•nucleotide complexes (1 μM HflX, 125 μM nucleotide final concentrations), the rate and extent of ribosome dissociation as reflected by the overall change in light scattering varied, as a function of the nucleotide present (Figure 6B). Light scattering time courses were best fit with a two exponential function. *Apo* HflX and HflX•GDP facilitated ribosome dissociation to the least extent (∼1.5% LS change, k_app1_ = 0.0069 ± 0.0002 s^−1^ and k_app2_ = 0.0038 ± 0.0002 s^−1^ for HflX•GDP). HflX•GDPNP dissociated ribosomes to a greater extent than HflX•GDP, but the rate of dissociation is approximately the same (∼20% LS change, k_app1_ = 0.017 ± 0.007 s^−1^ and k_app2_ = 0.0044 ± 0.0002 s^−1^). In contrast, HflX•GTP rapidly split 70S ribosomes to the greatest extent (∼30% LS change, k_app1_ = 0.065 ± 0.001 s^−1^ and k_app2_ 0.0018 ± 0.0001 s^−1^). The rate of ribosome dissociation by HflX•GTP is approximately equal to the rate of ribosome dissociation by EF-G•GTP and RRF (34).

To investigate a putative role for the C-terminal domain of HflX in 70S dissociation we created a C-terminal truncation variant at Lys372 (26). Light scattering experiments carried out using this truncation variant in the presence of GTP revealed HflX ΔL372 dissociated ribosomes to a much lower extent than wild-type HflX in the presence of GTP or GDPNP (∼10% LS change; Supplementary Figure S7A). As HflX dissociates the ribosome with a greater rate and extent in the presence of GTP compared to GDPNP, hydrolysis is required for optimal dissociation. Therefore, we tested the ΔL372 truncation variant for GTP hydrolysis finding that it is still active with a rate of 0.065 ± 0.006 μM s^−1^, compared to wild-type HflX, 0.103 ± 0.001 μM s^−1^ (Supplementary Figure S7B).

In order to address the questions if GTP hydrolysis is coupled to splitting of the subunits, we used a higher (30 mM) Mg^2+^ concentration to stabilize the 70S ribosome. Under these conditions, no dissociation of the subunits could be observed after rapid mixing, either in the presence or the absence of HflX (Supplementary Figure S8A). Also, inhibiting dissociation did not affect HflX binding (Supplementary Figure S8B–C) or the ribosome stimulated multiple turnover GTPase activity (Figure 4C–D), suggesting that indeed 70S ribosome is the target of HflX and that GTP hydrolysis occurs without the dissociation of the 70S ribosome.

## DISCUSSION

Our data demonstrate that HflX covalently crosslinks with ribosomal proteins and rRNA on the E-site side of the 70S ribosome in solution (Figures 2 and 3). The localization of HflX's binding site to the tRNA exit site of the 70S ribosome makes HflX the first known trGTPase to bind at that location and whose GTPase activity is stimulated by the ribosome. Classical trGTPases (EF-G, EF-Tu, RF3, LepA and others) bind to, and are stimulated by, the SRL located at the A-site side of the ribosome, opposite to where HflX binds the 70S ribosome. While the current manuscript was under review the cryo-EM structure of a 50S ribosomal subunit HflX complex was reported, locating HflX in the A-site of the 50S ribosomal subunit (38). The cryo-EM model shows the N-terminal HflX domain reaching into the PTC, overlapping with both the A- and P-site while the G-domain is oriented away from the SRL. This suggests a different mode by which GTPases can be activated by the ribosome. The idea that the ribosome possesses additional GTPase stimulatory centres is not novel, as ObgE has previously been shown to bind to a similar region of the 50S subunit (38,39). In addition, our data reported here demonstrate that HflX when binding to the 70S ribosome, interacts with the ribosomal E-site and that this interaction stimulates the intrinsic GTPase activity of HflX on the opposite side of the ribosome via a novel mode of activation.

Using a variant of HflX lacking the cysteine residue in the unresolved C-terminal domain (Figure 1B) labeled with AzP (HflX-C415L), no crosslinks were observed compared to the labeled wild-type enzyme after UV exposure (Supplementary Figure S3). Interestingly these results indicate that Cys415 can be within 11 Å of ribosomal proteins L2/S18 and L5 which are at least 60 Å apart (Figure 3A). The fact that Cys415 is able to crosslink with ribosomal proteins on either side of the E-site opening suggests that the C-terminal domain is flexible and able to move between these positions. The flexibility of the C-terminal domain could be attributed to HflX exploring different conformations while bound to the ribosome. This is consistent with fluorescence resonance energy transfer (FRET) experiments measuring the GDPNP affinity to HflX•70S complexes that showed two-exponential behavior, an initial binding step followed by a conformational change upon GDPNP binding (36). Additionally, we observed lower efficiency of crosslink formation in the GDPNP and GDP-bound states of HflX compared to *apo*, suggesting that nucleotide binding stabilizes HflX in a certain conformation required for its activity (Supplementary Figure S2).

Consistent with these observations is a model in which the N-terminal HflX-domain binds directly into the E-site, while the G-domain is bound to the outer surface of the ribosome resulting in the C-terminal domain being located at the periphery of the ribosome rendering it more solvent-exposed. This is supported by the fact that truncations of the N-terminal domain abolish interaction of HflX to the 70S ribosome and 50S/30S ribosomal subunits (26) and our previous limited proteolysis experiments showing increased protection of the N-terminus in the presence of the 70S and 50S ribosome particles (36).

In addition to the previously reported effect of chloramphenicol (36), we show that other PTC/PET binding antibiotics inhibit stimulation of HflX GTPase activity (Figure 4). The binding site of these antibiotics, when considered with HflX bound to the E-site, suggests a communication between the P- and E-sites of the ribosome. Previously, no communication to the E-site has been shown to stimulate GTPase activity of trGTPases as all the canonical protein factors bound to the A-site are activated by the GAC and SRL. Yet several proteins have been reported to interact with the E-site during translation, such as EttA. EttA interacts with the P-site tRNA and inhibits ribosome dynamics preventing translation in energy-depleted cells (40,41). To do this, EttA, while bound to the E-site, reaches into the P-site making direct contact with initiator tRNA^fMet^. Direct contact between a factor and a particular location on the ribosome is one potential communication pathway, yet the signal could also be relayed through the ribosome itself to the factor. Our proposed binding site for HflX in the E-site suggests that HflX makes direct contact with the P-site, specifically the PTC/PET.

Whether or not HflX can reach into the PTC/PET, our data show that even a small molecular inhibitor bound in the PTC/PET influences the ribosome-dependent stimulation of HflX's GTP hydrolysis activity. Interestingly, HflX is not activated to hydrolyze GTP by the conformational state of antibiotic-bound PTC/PET, which indicates that inhibition of HflX may be another previously overlooked mode of action of these antibiotics. Furthermore, other conformational states of the PTC/PET induced by ribosome bound partners are likely to stimulate HflX. During elongation, a growing polypeptide chain is most common in the PTC/PET and specific sequences of the polypeptide are able to influence the conformation of the PET (42,43). Work reported here on the ribosome-stimulation of HflX have all used vacant ribosomes purified from *E. coli* cells. There are two potential reasons why vacant ribosomes stimulate HflX: first, the functional role of HflX is to interact with empty 70S ribosomes, and second, vacant 70S ribosomes are able to explore several conformational states, one of which is the state that activates HflX and is also characteristic to a particular functional state of the ribosome. As GTP hydrolysis is typically tied to a GTPases functional role *in vivo*, understanding the conformational state of the ribosome that activates GTP hydrolysis is crucial to determining the function of HflX in the cell.

In order to address how these antibiotics inhibit HflX stimulation, we first wanted to see if HflX binding to the ribosome was impeded by the presence of these antibiotics (Figure 5). The fact that the presence of antibiotics did not interfere with HflX binding to the ribosome suggests that they might instead influence the nucleotide binding properties of the HflX•ribosome complex or influence the conformation of the stimulatory region of the ribosome involved in catalysis by HflX, much like the SRL does for classical trGTPases. Additional studies are required to determine the exact cause of inhibition.

Our initial experiment attempting to obtain a cryo-EM structure of HflX bound to the 70S ribosome revealed only 50S and 30S subunits, suggesting a ribosome splitting activity of the protein. Using light scattering experiments to indirectly observe 70S dissociation into 50S and 30S ribosomal subunits (Figure 6A) we found that HflX is indeed able to split the 70S ribosome in a nucleotide dependent manner (Figure 6B). The GTP-bound state of HflX very efficiently splits the ribosome compared to the GDP-bound and *apo* states, which show no detectable levels of ribosome dissociation. The use of the non-hydrolyzable analog of GTP, GDPNP, showed that hydrolysis is not required for splitting, yet splitting occurs at a much slower rate, indicating that hydrolysis is required for efficient splitting. These results differ from those of Zhang *et al*., who using a similar technique showed that HflX in the presence of GDPNP was more efficient at splitting compared to GTP (38). These conflicting observations could be due to differences in buffer conditions (HEPES-polymix compared to a Tris based buffer used in this report) or a contamination with GTP generated using the Pyruvate kinase/Phosphoenolpyruvate system present in their assay (38). However, more detailed mechanistic studies are required to reconcile this difference.

Furthermore, the truncation mutant of HflX lacking the C-terminal domain (HflX-ΔL372) was impaired in its ability to efficiently split the ribosome, indicating the importance of the C-terminal domain for ribosome splitting (Supplementary Figure S7A). As HflX splits ribosomes in the GTP-bound state we confirmed that HflX-ΔL372 is still capable to bind and hydrolyze GTP (Supplementary Figure S7B–C). Hydrolysis is unaffected by the C-terminal truncation, further supporting a functional role for the C-terminal domain in ribosome splitting. Interestingly, Zhang *et al*. show that in the 50S complex the C-terminal domain of HflX points out of the A-site and away from helix 69 (H69) of the 23S rRNA (intersubunit bridge B2a) which they propose is modulated by HflX to facilitate splitting (38). In contrast, our localization of the C-terminal domain in the E-site of 70S ribosome complex near the subunit interface between ribosomal proteins L2 and S18 (Figure 3 and Supplementary Figure S3) suggests that the C-terminus is positioned to insert between the two ribosomal subunits. This could contribute to dissociation, for example, by modulating the intersubunit bridges B7a and B2a which involves h23 of the 16S rRNA and H68 of the 23S rRNA as well as H69 and h44, respectively. Interestingly, the binding site of HflX on the 30S ribosomal subunit suggested by the crosslinking data reported here is very similar to the interaction site of initiation factor 3. As some degree of ribosome splitting is still observed in the absence of the C-terminal domain, the other domains of HflX are likely to also contribute to splitting, but the correct placement of the C-terminal domain is required for efficient splitting (Supplementary Figure S7A). Such an effect might be caused by preventing re-association of the subunits after dissociation through occlusion of parts of the interaction surface between the two ribosomal subunits by the bound HflX, consistent with the structure reported for the 50S•HflX complex (38).

In order to investigate if GTP-hydrolyis depends on the splitting activity we performed GTP-hydrolysis experiments in the presence of 30 mM Mg^2+^ which efficiently prevented ribosome dissociation by HflX•GTP (Supplementary Figure S8A). These conditions did not affect HflX binding to or GTPase stimulation by the ribosome (Figure 4A–B and Supplementary Figure S8B–C). Thus, ribosome splitting is not required for GTP hydrolysis to occur, suggesting a different mode of HflX regulation. Furthermore, this observation supports a binding site of HflX on the 70S ribosome that does not require stabilization by an altered intersubunit bridge as suggested by Zhang *et al*. (38).

**Figure 7.**
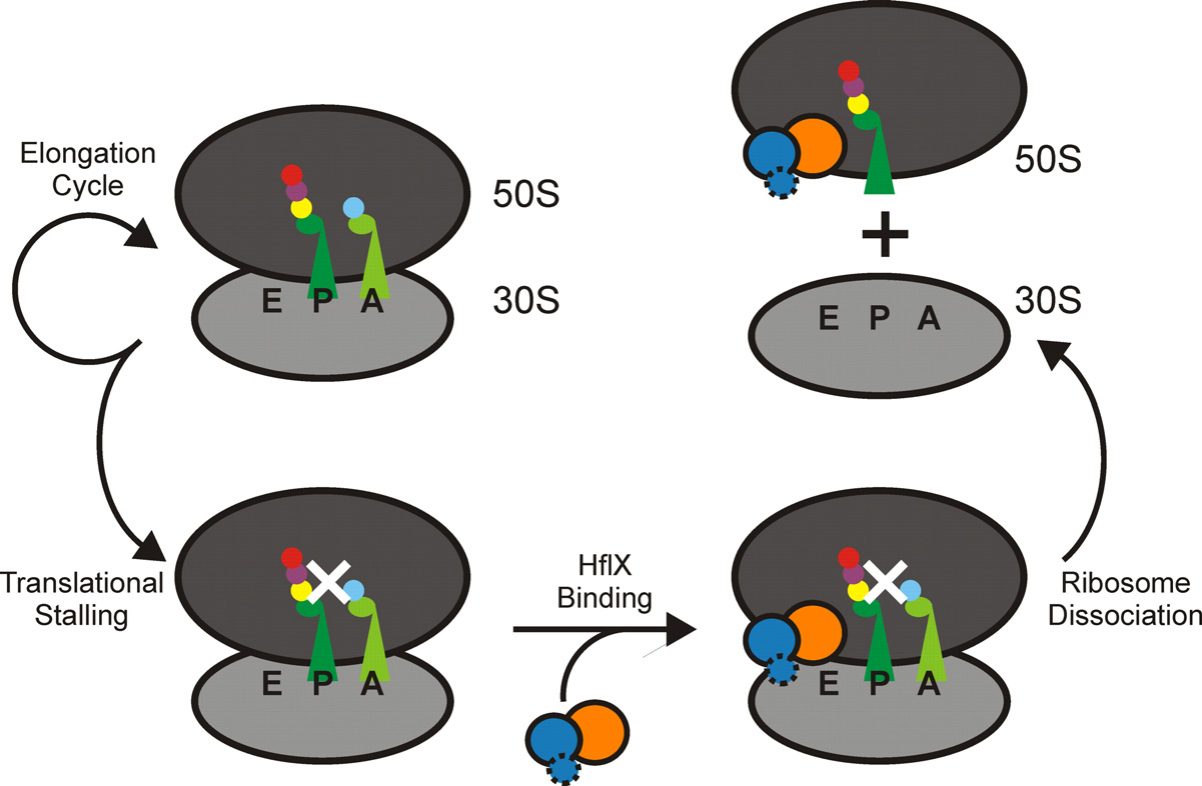
Proposed model of HflX function. Stalling of translation occurs during cellular stress, leaving the E-site vacant. HflX is able to bind to the vacant E-site to assess the state of the PTC/PET. Upon recognizing the ribosome is stalled, HflX hydrolyzes GTP and splits the 70S ribosome into 50S and 30S ribosomal subunits that subsequently can be used in another round of translation.

The location of the *hflX* gene downstream of *hfq* under the control of a heat sensitive promoter strongly suggests that HflX is required during heat stress, as confirmed by Zhang *et al*. (38). During cellular stress, translational arrest is common (44), leaving the E-site of the ribosome empty and the PTC/PET filled with the growing polypeptide chain attached to the P-site tRNA. We propose that HflX is able to bind to the empty E-site of a stalled ribosome, sense the stalled polypeptide in the PTC/PET which triggers HflX to hydrolyze GTP, and upon hydrolysis split the ribosome, freeing up the subunits to be used in another round of translation (Figure 7). As the A-site of a stalled ribosome is typically filled with an aminoacyl-tRNA and the E-site is left empty after the deacylated tRNA exits, it would be possible for HflX to bind into the E-site rather than the A-site. Following splitting of the ribosome, HflX could then bind to the A-site to prevent reassociation of the 50S and 30S ribosomal subunits and block the association of other trGTPases before reassembly of a new 70S complex. Further studies are required to determine the exact role of HflX during heat stress and the mechanism by which HflX is able to provide a fitness advantage to cells.

In conclusion, this work provides for the first time evidence that the ribosome-associated GTPase HflX is able to monitor the ligand induced state of the PTC, and that it catalyzes efficient splitting of the 70S ribosome in a nucleotide-dependent manner. Furthermore, our proposed location where HflX binds to the 70S ribosome, and the respective ribosome-dependent stimulation of GTP hydrolysis, indicate a previously unidentified GTPase activation site on the ribosome needed for efficient translation.

## Supplementary Material

SUPPLEMENTARY DATA
