# Supplementary material for: The conserved GTPase HflX is a ribosome splitting factor that binds to the E-site of the bacterial ribosome
Source: Nucleic Acids Res. 2016 Jan 4;44(4):1952–61. doi: 10.1093/nar/gkv1524 (PMC4770234; doi:10.1093/nar/gkv1524)
Supplement: SUPPLEMENTARY DATA [file supp_44_4_1952__index.html]

The conserved GTPase HflX is a ribosome splitting factor that binds to the E-site of the bacterial ribosome — The conserved GTPase HflX is a ribosome splitting factor that binds to the E-site of the bacterial ribosome — SUPPLEMENTARY DATA 

# The conserved GTPase HflX is a ribosome splitting factor that binds to the E-site of the bacterial ribosome

## SUPPLEMENTARY DATA

- SUPPLEMENTARY DATA
