# Supplementary material for: The conserved GTPase HflX is a ribosome splitting factor that binds to the E-site of the bacterial ribosome
Source: Nucleic Acids Res. 2016 Jan 4;44(4):1952–61. doi: 10.1093/nar/gkv1524 (PMC4770234; doi:10.1093/nar/gkv1524)
Supplement: SUPPLEMENTARY DATA [file supp_gkv1524_nar-02987-r-2015-File009.pdf]

Supplemental Material

for

The conserved GTPase HflX is a ribosome splitting factor that binds to the E-site of the bacterial ribosome

Coatham, M.L.*, Brandon, H.E.*, Fischer, J.J., Schümmer, T., and Wieden, H.-J.

*These authors contributed equally to this work.

Supplemental Figures

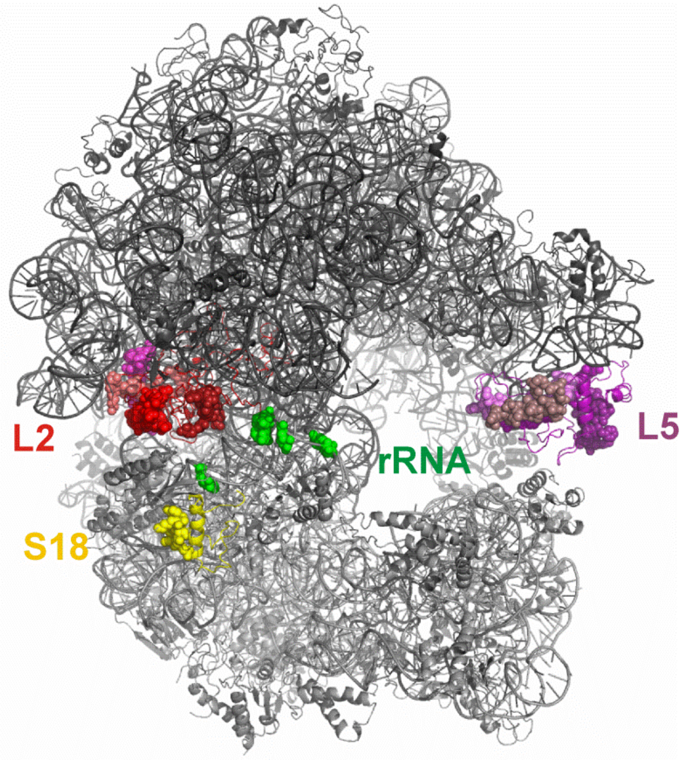

Supplemental Figure 1. Peptides identified by mass-spectrometry following crosslinking of the 70S ribosome with AzP-HflX. The respective ribosomal proteins identified by mass-spectrometry (listed in Supplemental Table 1) are shown on the 70S ribosome structure (PDB 4V4Q) as shades of red, purple, and yellow. Spheres indicate peptides identified matching L2, L5, and S18 respectively. The respective rRNA crosslinks are shown as green spheres.

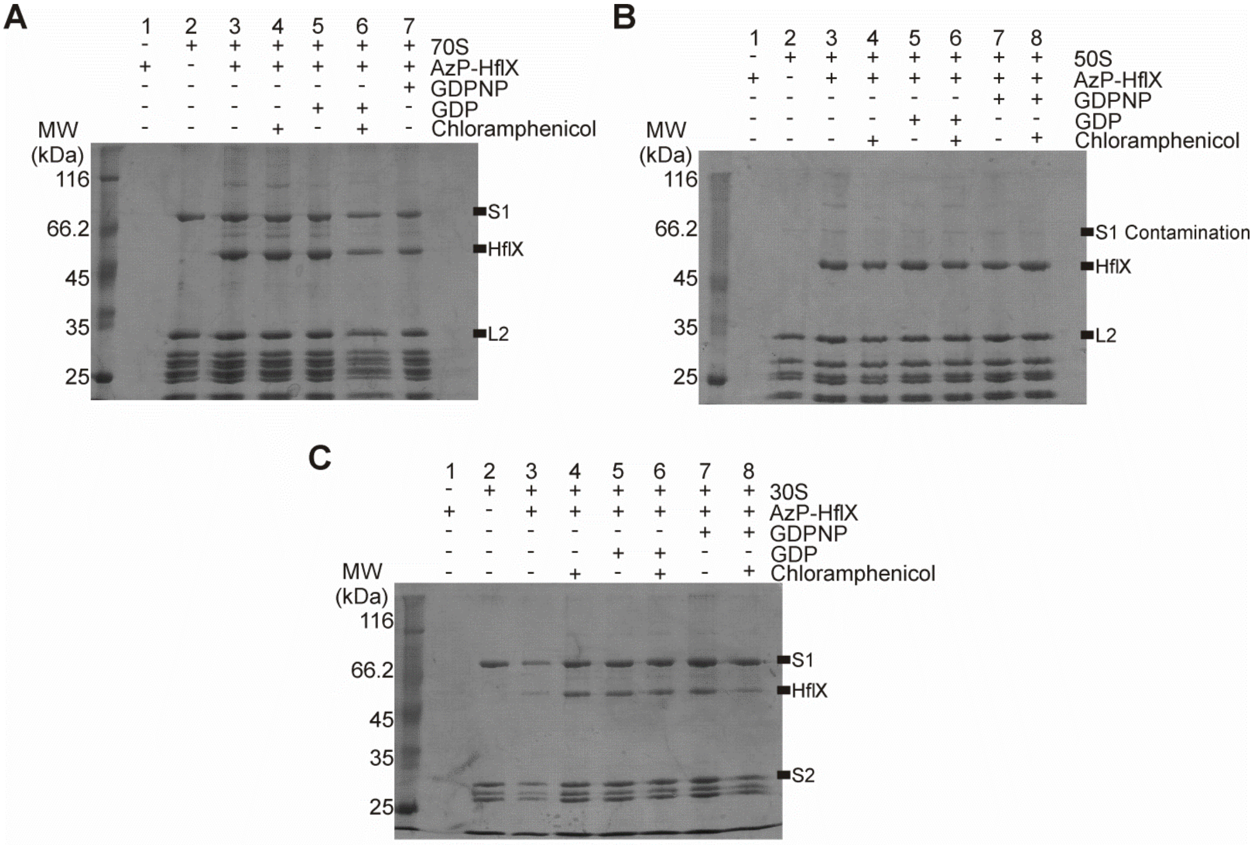

Supplemental Figure 2. Crosslinking of AzP-HflX to 70S ribosomes, and 50S/30S ribosomal subunits in different nucleotide bound states. AzP-HflX was incubated with (a) 70S ribosomes, (b) 50S and (c) 30S ribosomal subunits in the presence of no nucleotide (*apo)*, GDP, or GDPNP to test the effect of the nucleotide bound state on the crosslinking efficiency and pattern. Furthermore, no effect of a bound PTC binding antibiotic (chloramphenicol) was observed.

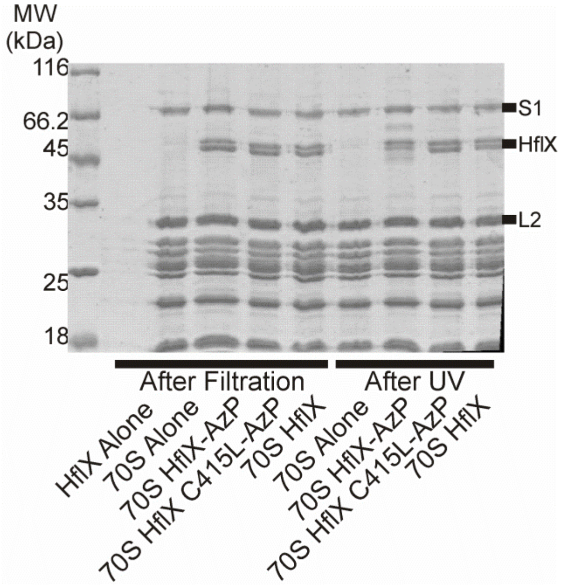

Supplemental Figure 3. Removal of the C-terminal cysteine in HflX (Cys415) abolishes crosslinks formed with AzP labeled protein. HflX C415L labeled with AzP bound to the 70S ribosome were subjected to microfiltration to remove unbound HflX C415L-AzP and subsequently analyzed by SDS-PAGE (before and after UV-exposure). No additional higher molecular weight bands are observed for HflX C415L-AzP or unlabeled wild-type HflX compared to those observed for wild-type AzP labeled HflX.

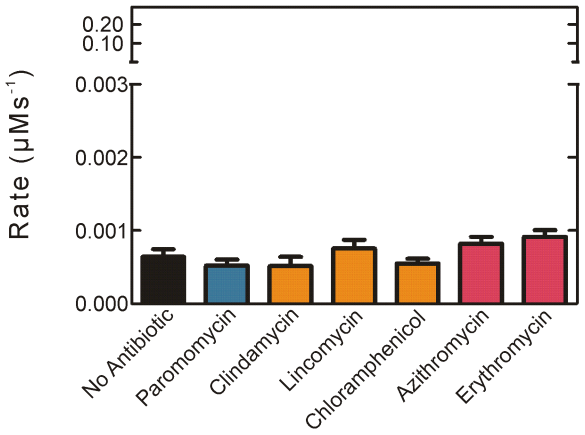

Supplemental Figure 4. Antibiotics do not inhibit intrinsic hydrolysis by HflX. HflX (1 μM) was incubated with [γ^{32}P]-GTP (125 μM) and antibiotic (500 μM). Reactions were quenched at successive time points and the amount of released [^{32}P] inorganic phosphate was quantified to determine the rate of hydrolysis. Antibiotics that target several regions of the ribosome were tested including those that bind to the decoding centre (teal), peptidyl transferase centre (orange), and peptide exit tunnel (pink).

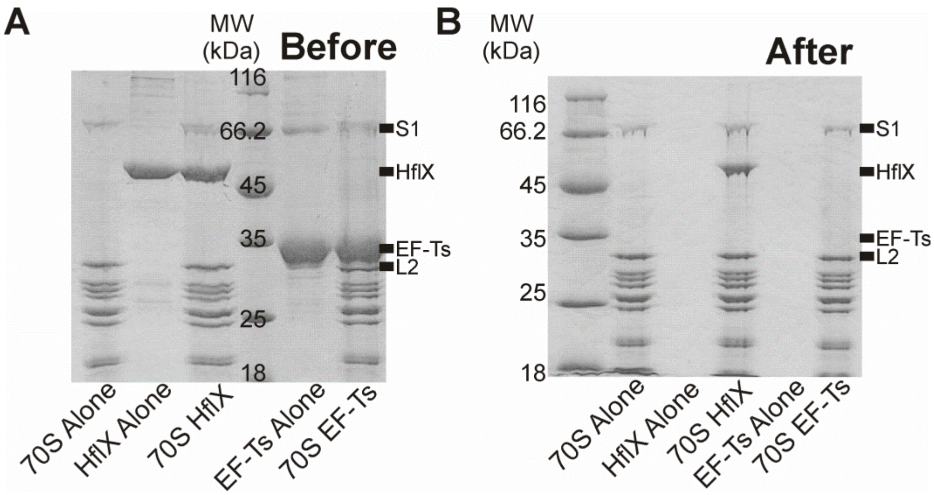

Supplemental Figure 5. Microfiltration experiments to confirm that ribosomes do not interfere with proteins passing through the filter. EF-Ts, the nucleotide exchange factor for EF-Tu, was used as it does not interact with the ribosome. EF-Ts or HflX (5 μM) was incubated with 70S ribosome (1 μM) before filtration. Samples of the (a) before and (b) after filtration solutions were analyzed by SDS-PAGE to verify correct stoichiometry and that HflX remained above the filter due to an interaction with ribosomes. EF-Ts was observed to not remain above the filter even in the presence of ribosomes.

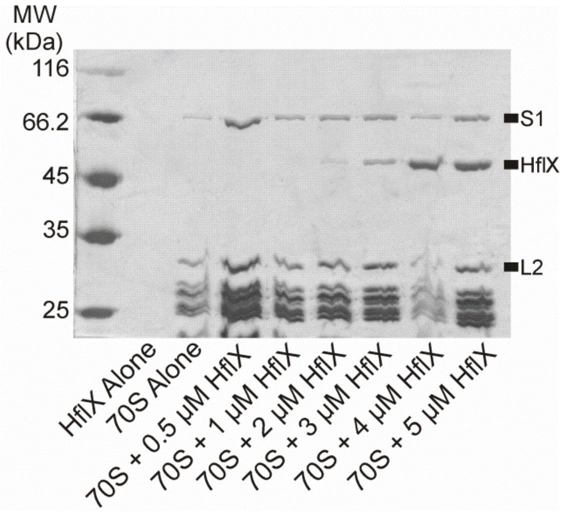

Supplemental Figure 6. Sucrose cushion ultracentrifugation experiments confirm HflX binding to the 70S ribosomes. Increasing amounts of HflX (0.5 – 5 µM) were incubated with 70S ribosomes (0.5 µM) prior to loading onto a 10% sucrose cushion. Following centrifugation in a MX 150 Sorvall micro-ultracentrifuge (Thermo Scientific) using an S140 AT rotor for 4 hours at 89 000 rpm, the supernatant was carefully removed before the pellets were resuspended in buffer and subsequently analyzed by SDS-PAGE and silver staining.

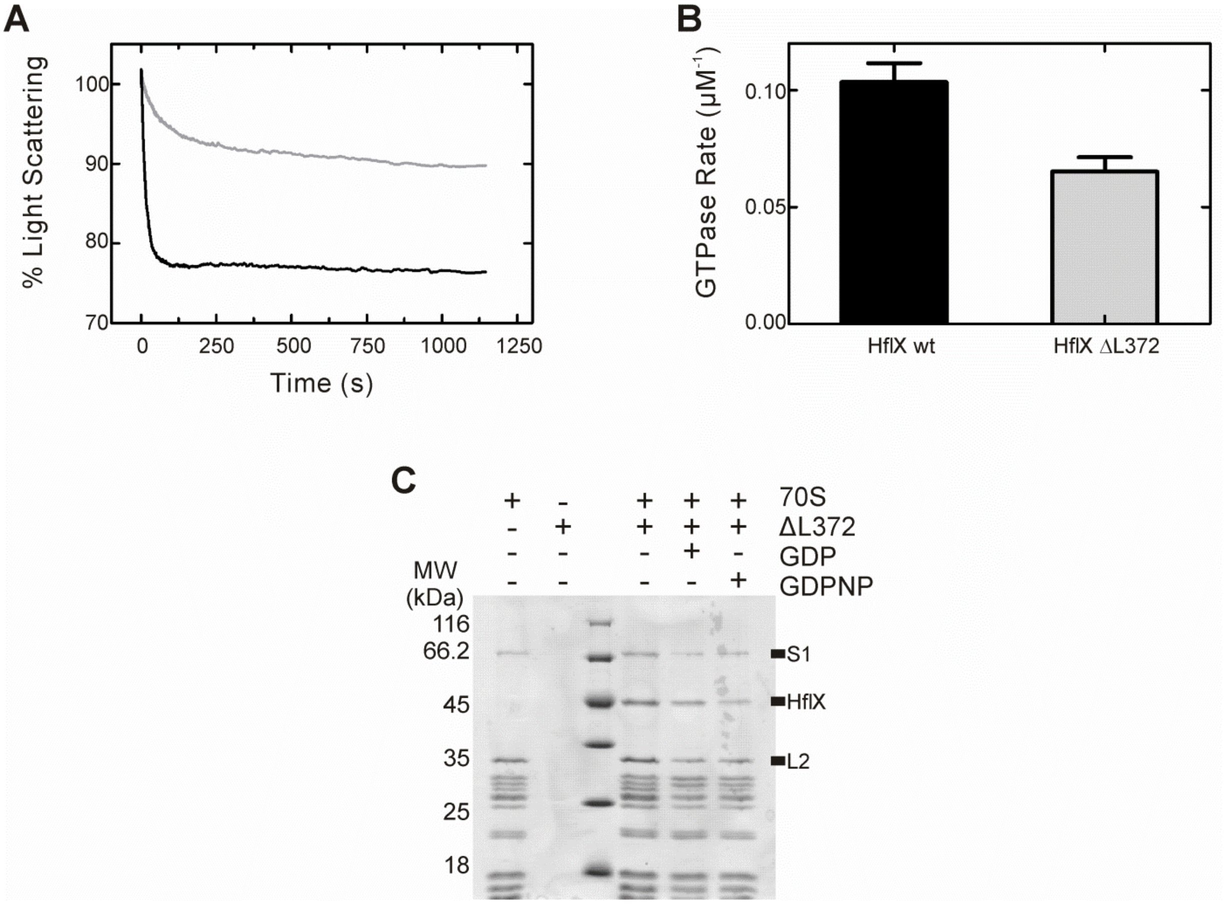

Supplemental Figure 7. The C-terminal domain of HflX is required for efficient ribosome splitting. (a) 70S dissociation experiments using HflX ΔL372•GTP (light grey) show a decrease in light scattering and a slower rate of dissociation compared to wild-type HflX (dark grey). (b) Rate of GTP hydrolysis by HflX ΔL372 is not affected by the removal of 55 amino acids at the C-terminus. (c) HflX ΔL372 is able to bind the 70S ribosome in all nucleotide bound states.

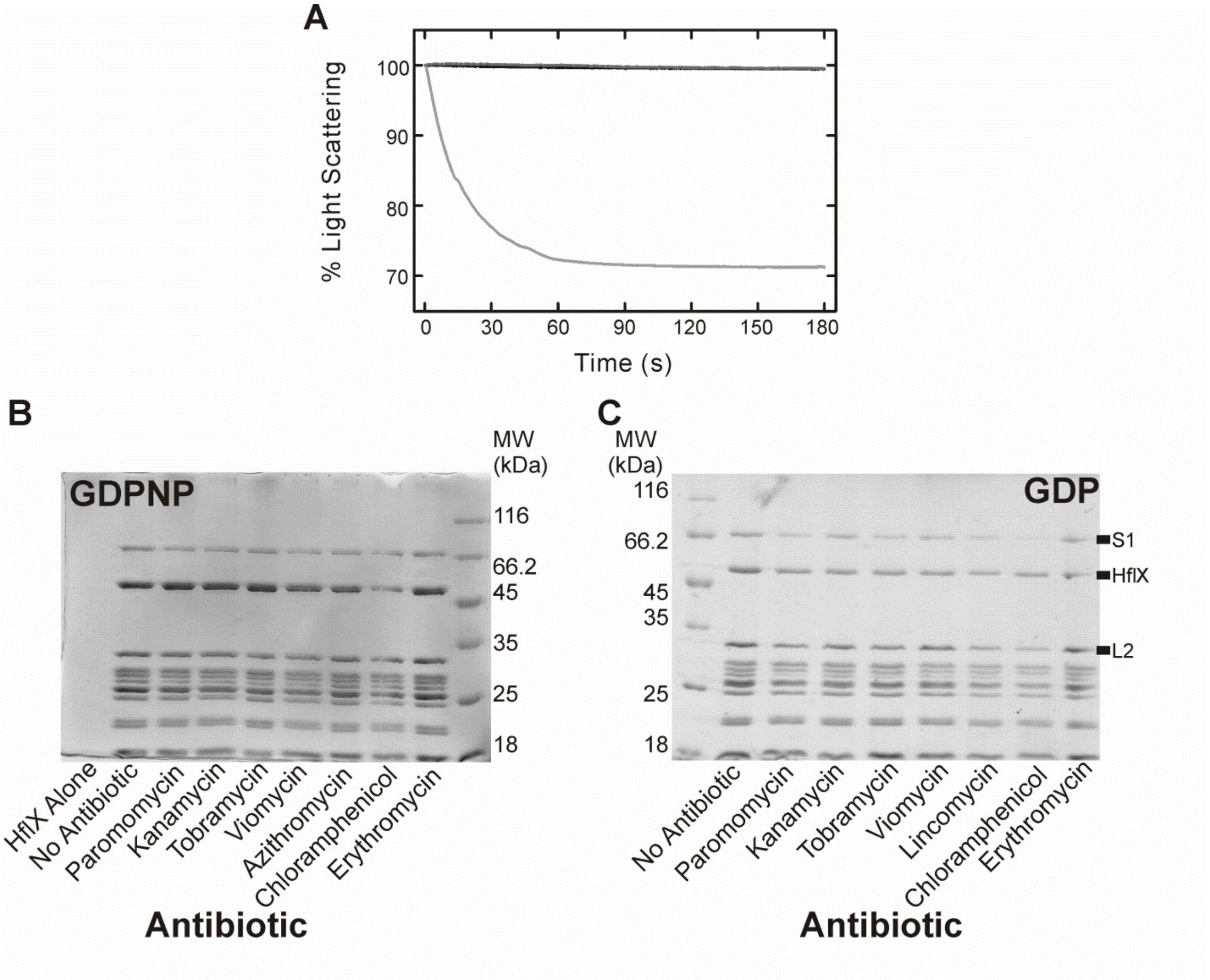

Supplemental Figure 8. Effect of 30 mM Mg^{2+} on ribosome splitting, binding, and GTPase stimulation of HflX. (a) 70S splitting in the presence of HflX•GTP was inhibited by the presence of 30 mM Mg^{2+} ($TAKM_{30}$, dark grey). Controls of 70S ribosomes in $TAKM_5$ buffer are mixed with either $TAKM_5$ buffer (black) or TAK buffer (light grey). GTPase inhibiting antibiotics do not interfere with binding of HflX to the 70S ribosome, either in the presence of 500 µM GDPNP (b) or 500 µM GDP (c). All experiments in b and c are carried out in the presence of $TAKM_{30}$ (50 mM Tris-Cl pH 7.5 at 4°C, 70 mM NH_4Cl, 30 mM KCl, 30 mM $MgCl_2$), preventing the dissociation of the 70S ribosomes into 50S and 30S subunits.

Supplemental Tables

Supplemental Table 1. List of peptides found by mass-spectrometry analysis.

Band Label	Peptide #	Sequence	Amino Acids	Color
**HflX***	HflX-1	KAVEIAEAVKA	62-72	
	HflX-2	RTGLILDIFAQRA	103-115	
	HflX-3	KLQVELAQLRH	121-131	
	HflX-4	RGPGETQLETDRRL	153-166	
	HflX-5	KADVPTVSLVGYTNAGKS	194-211	
	HflX-6	RVYAADQLFATLDPTLRR	221-238	
	HflX-7	RHLPHDLVAAFKA	257-269	
	HflX-8	RQATLLLHVIDAADVRV	274-291	
	HflX-9	KIDMLEDFEPRI	318-329	
	HflX-10	RLSGEVAGHTLRL	360-372	
	HflX-11	RFYQLQAIEKE	382-392	
		Percent Coverage HflX:	35.68%	

Band Label	Peptide #	Sequence	Amino Acids	Color
**HflX-L2**				
	L2-1	KGKPFAPLLEKN	26-37	pink
	L2-2	RSANIALVLYKD	87-98	light red
	L2-3	KAGDQIQSGVDAAIKPGNT	111-129	red
	L2-4	RDGAYVTLRL	167-176	dark red
		Percent Coverage L2:	19.41%	
	HflX-1	KAVEIAEAVKA	62-72	
	HflX-2	RTGLILDIFAQRA	103-115	
	HflX-3	KLQVELAQLRH	121-131	
	HflX-4	KADVPTVSLVGYTNAGKS	194-211	
	HflX-5	RVYAADQLFATLDPTLRR	221-238	
	HflX-6	RHLPHDLVAAFKA	257-269	
	HflX-7	RQATLLLHVIDAADVRV	274-291	
	HflX-8	KIDMLEDFEPRI	318-329	
	HflX-9	RLSGEVAGHTLRL	360-372	
	HflX-10	RFYQLQAIEKE	382-392	
		Percent Coverage HflX:	32.39%	

Band Label	Peptide #	Sequence	Amino Acids	Color
**HflX-L5**				
	L5-1	KITLNMGVGEAIADKKL	33-49	lightest purple
	L5-2	KLLDNAAADLAAISGQKP	48-65	light purple
	L5-3	RGLDITITTTAKS	150-162	purple
	L5-4	RALLAAFDFPFRK	167-179	dark purple
		Percent Coverage L5:	34.08%	
	HflX-1	RTGLILDIFAQRA	103-115	
	HflX-2	RQATLLLHVIDAADVRV	274-291	
		Percent Coverage HflX:	7.28%	

Band Label	Peptide #	Sequence	Amino Acids	Color
**HflX-S18**				
	S18-1	RFTAEGVQEIDYKD	12-25	yellow
		Percent Coverage S18:	18.67%	
	HflX-1	RYDAGEQAVLVHI	4-16	
	HflX-2	KAVEIAEAVKA	62-72	
	HflX-3	KATGASVVLFDHALSPAQERN	71-91	
	HflX-4	RTGLILDIFAQRA	103-115	
	HflX-5	KLQVELAQLRH	121-131	
	HflX-6	KADVPTVSLVGYTNAGKS	194-211	
	HflX-7	RVYAADQLFATLDPTLRR	221-238	
	HflX-8	RHLPHDLVAAFKA	257-269	
	HflX-9	RQATLLLHVIDAADVRV	274-291	
	HflX-10	KIDMLEDFEPRI	318-329	
	HflX-11	RLSGEVAGHTLRL	360-372	
	HflX-12	RFYQLQAIEKE	382-392	
		Percent Coverage HflX:	40.38%	
